# MIKC-type MADS-box transcription factor gene family in peanut: Genome-wide characterization and expression analysis under abiotic stress

**DOI:** 10.3389/fpls.2022.980933

**Published:** 2022-10-20

**Authors:** Yifei Mou, Cuiling Yuan, Quanxi Sun, Caixia Yan, Xiaobo Zhao, Juan Wang, Qi Wang, Shihua Shan, Chunjuan Li

**Affiliations:** Key Laboratory of Peanut Biology, Genetic & Breeding, Ministry of Agriculture and Rural Affairs, Shandong Peanut Research Institute, Qingdao, China

**Keywords:** MADS-box transcription factor, phylogenetic analysis, expression patterns, abiotic stress, peanut

## Abstract

Peanut (*Arachis hypogaea*) is one of the most important economic crops around the world, especially since it provides vegetable oil and high-quality protein for humans. Proteins encoded by MADS-box transcription factors are widely involved in regulating plant growth and development as well as responses to abiotic stresses. However, the MIKC-type MADS-box TFs in peanut remains currently unclear. Hence, in this study, 166 MIKC-type MADS-box genes were identified in both cultivated and wild-type peanut genomes, which were divided into 12 subfamilies. We found a variety of development-, hormone-, and stress-related *cis*-acting elements in the promoter region of peanut MIKC-type MADS-box genes. The chromosomal distribution of peanut MADS-box genes was not random, and gene duplication contributed to the expansion of the MADS-box gene family. The interaction network of the peanut AhMADS proteins was established. Expression pattern analysis showed that AhMADS genes were specifically expressed in tissues and under abiotic stresses. It was further confirmed *via* the qRT-PCR technique that five selected AhMADS genes could be induced by abiotic and hormone treatments and presented different expressive profiles under various stresses. Taken together, these findings provide valuable information for the exploration of candidate genes in molecular breeding and further study of AhMADS gene functions.

## Introduction

The MADS-box gene family, one of the largest transcription factors (TFs), has been widely found in fungi, animals, and plants (Shore and Sharrocks, 1995) and plays important roles in a wide variety of physiological and developmental processes (Alvarez-Buylla et al., 2000; Gramzow and Theissen, 2010). The MADS is an acronym for *minichromosome maintenance 1* (*MCM1*) (Passmore et al., 1989), *agmous* (*AG*) (Riechmann and Meyerowitz, 1996), *deficiens* (*DEF*) (Sommer et al., 1990), and *serum response factor* (*SRF*) (Norman et al., 1988) genes, and these four genes are from yeast, *Arabidopsis*, *Antirrhinum majus*, and humans, respectively. MADS-box genes contain a highly conserved MADS domain at the DNA-binding domain, and this domain can recognize the CArG-box in the *cis*-regulatory regions of target genes (Messenguy and Dubois, 2003).

Based on the structure of the conserved domains, MADS-box genes in plants can be divided into two major groups: type I and type II (Alvarez-Buylla et al., 2000). Type I MADS-box proteins possess only the MADS domain (M), and type II MADS-box proteins have a typical domain structure, which includes the M domain, intervening (I) domain, K domain, and C-terminal domain (Smaczniak et al., 2012). As a result of their characteristic domain structure, type II proteins are also known as MIKC-type MADS-box proteins (Kaufmann et al., 2005). MIKC-type genes can be further divided into MIKC^C^ and MIKC* according to the difference in the I domain (Henschel et al., 2002). MIKC-type MADS-box genes can be divided into 12 subfamilies based on the maximum likelihood phylogenetic tree of all MIKC-type MADS-box genes from *Arabidopsis thaliana*, rice (*Oryza sativa*), and wheat (Gramzow & Theissen, 2015). There is little information about type I MADS-box gene function, and their function appears only related to plant reproduction, particularly in female gametophyte, embryo, and seed development (Bemer et al., 2010; Masiero et al., 2011). However, the function of the type II MADS-box has been well documented, and it was proved to function in plant development, such as the morphogenesis of flower, root and seed morphogenesis, and embryo development (Parenicová et al., 2003; Silva et al., 2016; Malabarba et al., 2017). Moreover, the type II MADS-box was essential for the responses to different stresses (Yu et al., 2014; Jia et al., 2018; Zhao et al., 2020). Therefore, the identification and characterization of MIKC-type MADS-box genes are crucial for better understanding their biological functions as well as for providing references for breeding stress-resistant plants.

In recent years, MIKC-type MADS-box proteins have been identified and functionally characterized in a wide range of plant species, including *Arabidopsis thaliana* (Parenicová et al., 2003), tomato (Wang et al., 2019), rice (Arora et al., 2007), maize (Zhang et al., 2012), wheat (Schilling et al., 2019), and sheepgrass (Jia et al., 2018). The MADS-box gene was first identified in flower mutants of *A. majus* (Sommer et al., 1990) and *Arabidopsis* (Yanofsky et al., 1990). Further investigation showed that most plant MIKC-type MADS-box genes were closely related to flower morphogenesis, and the well-known ABCDE model for the development of floral organs was established. For example, *APETALA1* (*AP1*) is an A-class gene and is essential for sepal and petal development (Mandel et al., 1992); the B-class genes *AP3* and *PISTILLATA* (*PI*) are expressed in petals and stamens (Ng and Yanofsky, 2001); the *AG* is a C-class gene that regulates stamens and carpels as well as functions in preventing the indeterminate growth of the floral meristem (Ng and Yanofsky, 2001); *STK* is a D-class gene; and *SEPALLATA1*–*4* (*SEP1*–*4*) are E-class genes (Pinyopich et al., 2003). Some MIKC genes have been found to control different regulatory steps, such as four MADS-box genes *Flowering Locus c* (*FLC*) (Reeves et al., 2007), *Suppressor of Overexpression of Constans1* (*SOC1*) (Lee, 2010), *AGAMOUS-LIKE GENE 15/18* (*AGL15/18*) (A Da Mczyk et al., 2010), and *Short Vegetative Phase* (*SVP*) (Hartmann et al., 2000) as regulators of flowering time; seed pigmentation genes TRANSPARENT TESTA16 (*TT16*) (Nesi, 2002); bud dormancy-associated MADS-box genes (*DAM*) (Traver et al., 2020); and root development genes *Arabidopsis NITRATE REGULATED1* (*ANR1*), *AGL12/14*, and *AGL21* (Alvarez-Buylla et al., 2019, Tapia-Lopez et al., 2008). Moreover, MADS-box genes function in response to various stresses. In *Arabidopsis*, *AGL16* negatively participates in drought resistance by altering stomatal density, stomatal movement, and leaf ABA accumulation (Zhao et al., 2020). *AGL21*-overexpressing plants were more sensitive to ABA, salt, and osmotic stresses, while the mutants were less sensitive (Yu et al., 2017). In tomato, *SIMBP8* could be induced by MeJA, salt, high temperature, wounding and dehydration, and the RNAi plants were more tolerant to drought and salt stresses than WT plants (Yin et al., 2017). In addition, tomato *SIMBP11*-RNAi plants were sensitive to salt stress, and overexpressing plants exhibited higher tolerance to salt stress than WT plants (Guo et al., 2016). The functions of a large number of MADS-box genes have been identified in other plants, and the peanut MADS-box gene family and potential roles remain unclear.

Therefore, to elucidate the peanut MADS-box gene evolution and provide foundations for biological function research of this TF family, we conducted a comprehensive view of MADS-box genes using the released genome of two cultivars [Tifrunner (Bertioli et al., 2019) and shitouqi (Zhuang et al., 2019)] and two diploid ancestors of tetraploid peanut *A. duranensis* and *A. ipaensis* (Bertioli et al., 2016). A total of 186 MIKC-type MADS-box genes were identified in the peanut genome, then the phylogenetic relationships, gene structure, protein motifs, and *cis*-acting elements in the promoter were analyzed. Furthermore, the gene duplication, chromosomal locations, interaction networks, expression patterns, and potential roles in abiotic stresses were systematically investigated. These results can provide a theoretical and technical basis for better understanding this TF family, thereby facilitating the exploration of the function of MADS-box genes.

## Methods

### Identification of MADS-box genes

All the protein sequences of *A. thaliana* and *Arachis hypogaea* were obtained from public databases [*A. thaliana* from EnsemblPlants: http://plants.ensembl.org/index.html, *A. hypogaea* (cultivar “Tifrunner”) from PeanutBase: https://www.peanutbase.org/, and *A. hypogaea* (cultivar “shitouqi”) from http://peanutgr.fafu.edu.cn/index.php (Zhuang et al., 2019)]. The amino acid sequences of the diploid ancestors *A. duranensis* and *A. ipaensis* of cultivated peanut were all downloaded from PeanutBase. The HMM profile of SRF-TF (PF00319) was downloaded from the Protein family database (Pfam) (http://pfam.xfam.org/). The hmmsearch tool of HMMER3.0 software (http://hmmer.org) was used to search the local protein database to identify putative MADS-box proteins (E-value, 10^-10^). We confirmed the obtained protein sequences using the tools Pfam (http://pfam.xfam.org/), SMART (http://smart.embl-heidelberg.de/), and NCBI Batch CD-search (https://www.ncbi.nlm.nih.gov/Structure/bwrpsb/bwrpsb.cgi).

### Phylogenetic analysis

Protein sequences of peanut MADS-box genes and AtMADSs were aligned using the ClustalW tool (MEGA X software). The GBlocks tool (http://molevol.cmima.csic.es/castresana/Gblocks_server.html) was used to select the conserved blocks of the above multiple alignment, which eliminated divergent and poorly aligned regions. A maximum-likelihood (ML) phylogenetic tree was constructed with 1,000 bootstrap replications using MEGA X (Sudhir et al., 2018). Then, the constructed tree was visualized and annotated with particular colors in different subfamilies by EvolView (http://www.evolgenius.info/evolview/).

### Gene structure, protein conserved motif analysis, and prediction of functional interacting networks

Coding sequences (CDSs) and genome sequences of peanut *MADS* analyzed for gene structure were obtained from PeanutBase (https://www.peanutbase.org/). The gene structure of peanut MADS-box was visualized using the Gene Structure Display Server (GSDS) (http://gsds.cbi.pku.edu.cn/). Conserved motif analysis of the protein sequences was performed by MEME (http://meme-suite.org/) with the following parameters: the number of motifs was selected as 10, the minimum width of motifs was 6, and the maximum width of motifs was 50 (Bailey et al., 2009). The functional interacting networks of MADS-box proteins were analyzed using STRING (version 11.0) (Damian et al., 2018).

### Chromosomal localization and gene duplication

The location information of the MADS-box gene family on peanut chromosomes and chromosomal length were extracted from the GFF file in PeanutBase (https://www.peanutbase.org/). Gene location was visualized *via* the online tool MG2C v2.1 (http://mg2c.iask.in/mg2c_v2.1/index.html). The Multiple Collinearity Scan toolkit (MCScanx) (http://chibba.pgml.uga.edu/mcscan2/) was used to analyze gene duplication events (segmental and tandem duplications) of MADS-box genes, and potential homologous gene pairs (E-value <1e-5) were identified within the peanut genome. Segmental duplicated genes were considered to be generated through polyploidy and experienced chromosome rearrangements (Yu et al., 2005). Tandem duplications were defined as two or more adjacent homologous genes located on one chromosome without any intervening gene (Zhu et al., 2014). The results were displayed using TBtools software (Chen et al., 2020).

### Prediction of *cis*-acting elements in promoter sequences

The 1,500-bp sequences upstream of the translational initiation codon were analyzed as promoters to predict *cis*-acting elements. All promoter sequences were obtained from PeanutBase (https://www.peanutbase.org/) and submitted to PlantCARE to identify *cis*-acting elements (http://bioinformatics.psb.ugent.be/webtools/plantcare/html/). The location and function of various *cis*-elements were used to generate the figure through TBtools software (Chen et al., 2020).

### Analysis of MADS-box gene expression patterns in peanut

We obtained the fragments per kilobase of transcript per million fragments (FPKM) values of 22 tissues from PeanutBase (https://www.peanutbase.org/). The RNA-seq data of MADS-box genes under drought (Zhao et al., 2018) and salt (Zhang et al., 2020) stresses were analyzed from our previous work. All the obtained values were log2-transformed and visualized by Heml software (Deng et al., 2014).

### Plant materials and treatments

The *A. hypogaea* cultivar huayu71 was grown with a 16-h light and 8-h dark cycle at 20°C in a controlled climate chamber. The seedlings of three-leaf-stage plants were separately dipped into 20% PEG6000 or 200 mM NaCl for drought or salt treatment, respectively, and the control plants were dipped into water. Leaves and roots were harvested 0 h, 1 h, 6 h, 12 h, 24 h, and 48 h after treatment, and frozen in liquid nitrogen for further qRT-PCR analysis. Experiments were performed with three biological replicates for each stress treatment.

### RNA isolation and quantitative real-time PCR

The expression levels of three genes in two tissues and different stress treatments were analyzed by qRT-PCR. Total RNA was extracted using the Takara RNA Extraction Kit (Code No. 9767, TaKaRa, Dalian) following the manufacturer’s instructions. We used the Takara PrimeScript RT Reagent Kit (Code No. RR037, Takara, Dalian) to synthesize first-strand cDNA. qRT-PCR was completed with an ABI 7500 Fast machine (ABI, USA). The 20-μl reaction system was composed of 10 μl of TB Premix Ex Taq Mix (No. RR820, Takara, Dalian), 2 μl (100 ng) of cDNA, 0.8 μl of forward primer (5.0 μM), 0.8 μl of reverse primer (5.0 μM), and 6.4 μl of RNase-free water. The relative expression levels of each gene were calculated using the 2^−ΔΔCT^ method.

## Results

### Identification and characterization of MADS-box TFs in peanut

A total of 97 (cultivar “Tifrunner”), 89 (cultivar “shitouqi”), 53 (*A. duranensis*), and 58 (*A. ipaensis*) MIKC-type TFs in peanut were identified on the basis of the released peanut genome using the hmmsearch tool of HMMER3.0 software. The HMM profile of the MADS-box domain used in the BlastP search was PF00319. All the obtained MADS-box genes were further analyzed by NCBI and SMART website to confirm the whole MADS-box domain. Finally, 93 (cultivar “Tifrunner”), 83 (cultivar “shitouqi”), 45 (*A. duranensis*), and 48 (*A. ipaensis*) proteins were identified and named AhMADS1 to AhMADS93, AdMADS1 to AdMADS45, and AiMADS1 to AiMADS48.

The length of amino acids encoded by MADS-box genes ranged from 66 (AhMADS44) to 572 (AiMADS1), and the molecular weight (MW) ranged from 7.63 kDa (AhMADS44) to 63.93 kDa (AiMADS1). The predicted isoelectric point (pI) ranged from 4.49 (AhMADS77) to 10.57 (AiMADS26). Detailed information, including the above amino acid length, MW, gene ID, pI, and chromosome location, is given in **Table S1**.

### Phylogenetic analysis of the MADS-box TFs

**Figure 1:**
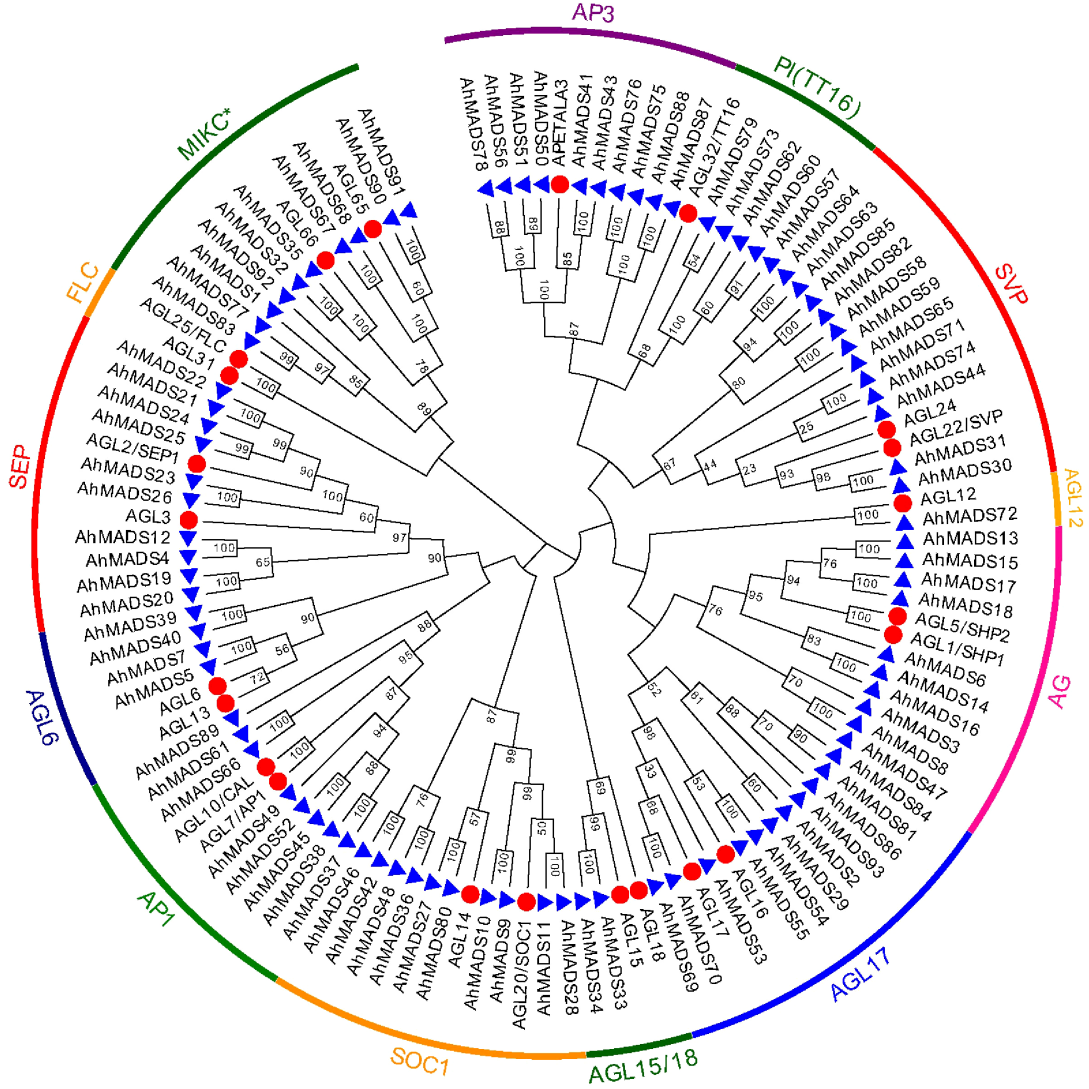
Phylogenetic tree of the peanut MIKC-type MADS-box gene family. Phylogenetic tree of 116 MADS-box proteins from peanut (93) and *Arabidopsis* (23). Twelve subfamilies are highlighted with specific colors.

To understand the phylogenetic relationships of peanut MADS-box proteins, two unrooted trees [93 peanut AhMADS with 23 *Arabidopsis* AtMADS TFs (**Figure 1**) and 93 genes of diploid ancestors with 23 *Arabidopsis* AtMADS TFs (**Figure S1**)] were constructed using MEGA X (maximum likelihood method). The genes were classified into two groups, MIKC^C^ and MIKC*, and the MIKC^C^ group was further subdivided into 12 major subfamilies [AG, AGL6, AGL12, AGL15/18, AGL17, AP1, AP3, FLC, PI (TT16), SEP, SOC1, and SVP] (**Figure 1**, **Table S1**, and **Figure S1**). The SVP subfamily contained the largest number (12) of MIKC-type AhMADS genes, followed by AGL17 with 11 genes. We found five subfamilies, SEP, SOC1, AG, AP3, and MIKC*, which each consisted of 10 MADS-box genes. The AGL6, AGL12, AGL15/18, and PI (TT16) subfamilies contained 4, 1, 2, and 5 genes, respectively, and the FLC subfamily contained no peanut genes. Unlike cultivated peanut, there are different in gene numbers of AGL12 and SVP subfamilies in wild peanut. More genes in AGL12 and SVP subfamilies were found in the diploid ancestors than cultivated peanut.

### Conserved motif and gene structure of the *AhMADS* TFs

**Figure 2:**
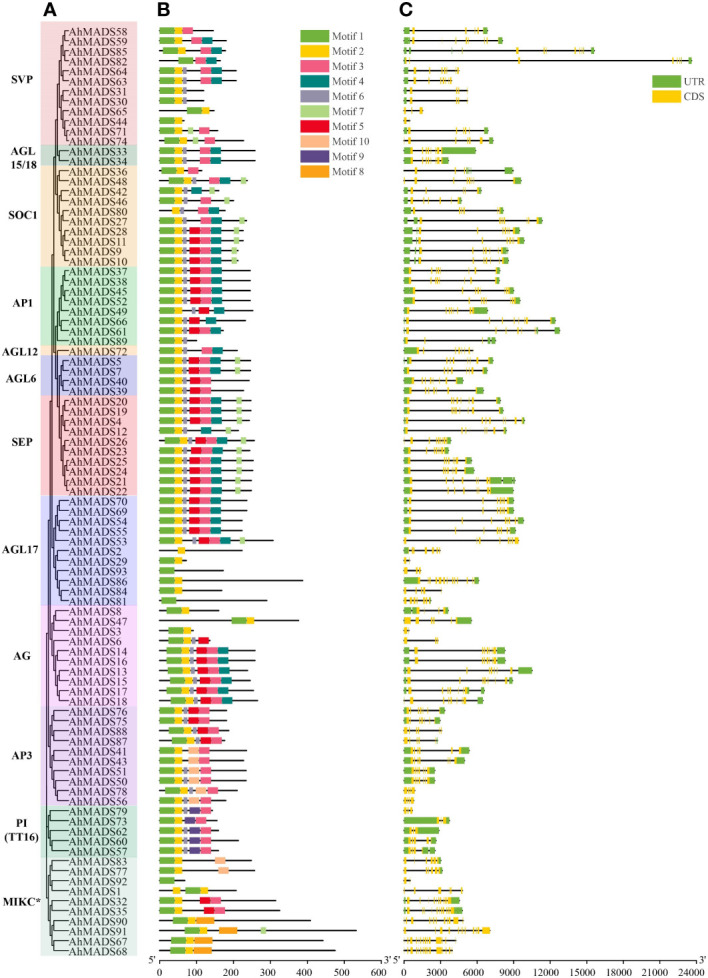
Phylogenetic relationship, conserved motifs, and exon−intron structure analysis of the MADS-box genes in peanut. **(A)** The maximum-likelihood phylogenetic tree was constructed using MEGA X with 1000 replicates. **(B)** Conserved motifs of MADS-box proteins. Ten conserved motifs are shown in different-colored boxes. **(C)** Exon−intron structures of MADS-box genes. The yellow boxes represent exons, and the black lines represent introns.

To characterize the protein structure in the peanut *MADS-box* family, the amino acid sequences of 93 AhMADS, 48 AiMADS, and 45 AdMADS were submitted to MEME to predict the conserved motifs. Ten conserved motifs, named motifs 1–10, were predicted in the peanut MADSs (**Figure 2**, **Figure S2**, **Table S3**). The MADS- and K-domains are the key determinants of DNA binding and protein dimerization (Alvarez-Buylla et al., 2000). Motif 1 and motif 2 present typical MADS domains, and most proteins had these two motifs, while four genes (AhMADS81, AhMADS82, AhMADS92, and AhMADS93) had only motif 1, three genes (AhMADS1, AhMADS2, and AhMADS80) had only motif 2, and a total of seven genes (7.5%) had incomplete MADS domains. A total of 19 out of 93 (20%) peanut MIKC-type MADS-box genes lacked a K-domain, which corresponded to motif 3. Compared with cultivated peanut, approximately 30% MADS-box genes from two diploid ancestors lacked motif 1 (**Figure S2**) and the proportion was much higher than that of cultivated peanut.

Gene structure analysis contributes to realizing gene family evolution. We investigated the exon−intron structures of MADS-box genes and found that the number of exons ranged from 2 (AhMADS3) to 16 (AhMADS68) (**Figure 2**, **Table S4**). A large number of genes (62 out of 93, 67%) contain six to nine exons. The lowest number of exons was identified in subfamilies AG, AGL17, SVP, and MIKC*, and a larger number of exons was found in subfamilies AGL17 (14), SVP (12), and MIKC* (11, 13, 14, 15, 16), coincidentally. It is noteworthy that most genes in the same subfamily (such as AGL15/18, AGL6, AP1, AP3, PI/TT16, SEP, and SOC1) showed similar exon numbers, implying that the functions of these genes in the same subfamily were similar.

### Interaction network of MADS-box TFs between peanut and *Arabidopsis*

**Figure 3:**
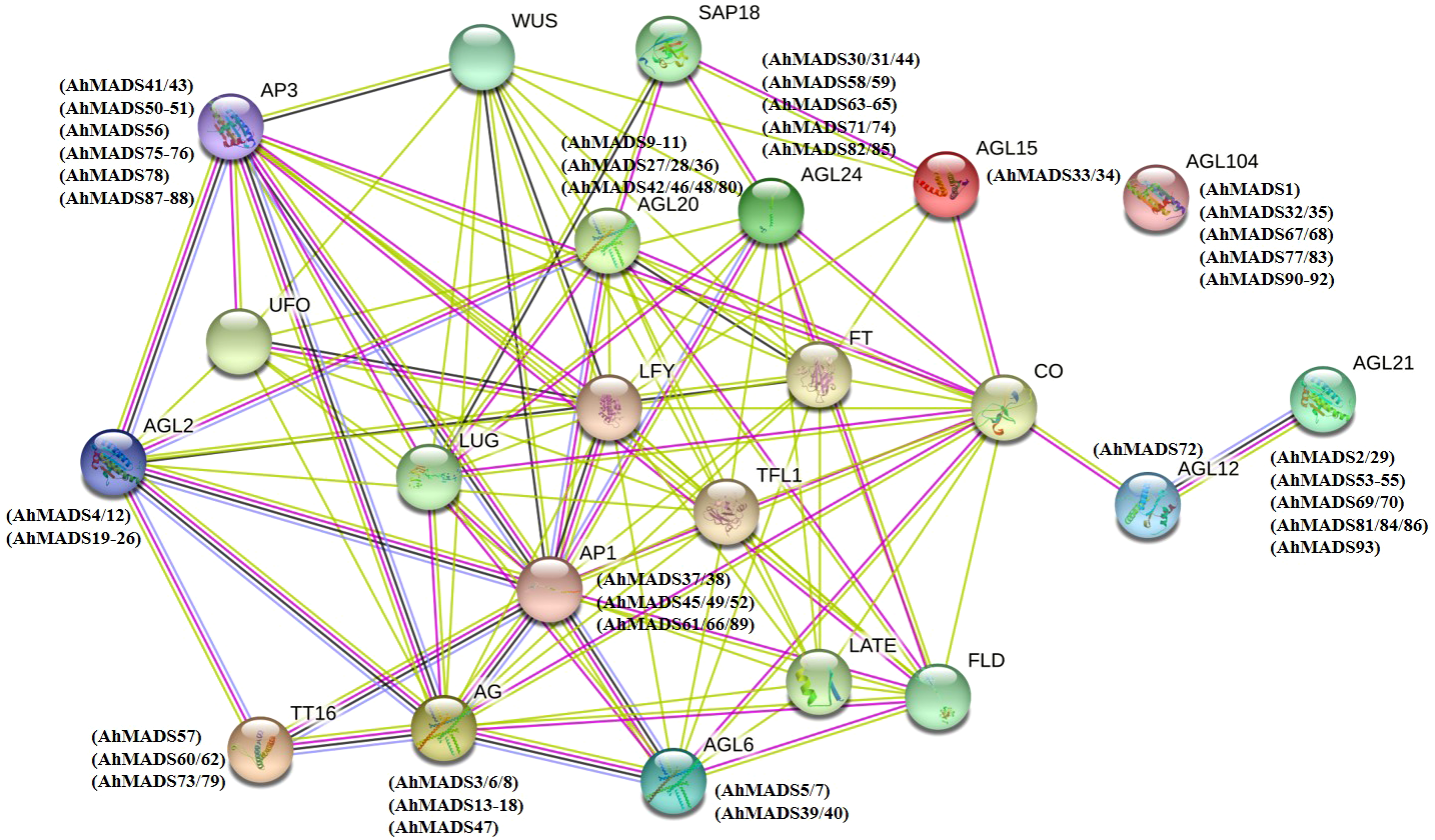
Interaction networks of AhMADS in peanut based on Arabidopsis data.

Protein−protein interactions are fundamental to cellular functions and provide valuable information for understanding plant biological processes (Gong et al., 2021). The STRING database integrates all known and predicted physical interactions and functional associations between proteins (Damian et al., 2020). We used STRING to predict the protein−protein interactions between peanut and *Arabidopsis* and constructed the interaction network of MADS-box genes (**Figure 3**). The network nodes represent proteins, and different color edges represent known or predicted protein−protein interactions. The protein sequence of AGL15 showed the highest similarity to the two AhMADS TFs (AhMADS33 and AhMADS34) and acted with the histone deacetylase complex subunit SAP18, which is involved in the regulation of salt stress. Moreover, AGL15 could interact with CO (B-box type zinc finger protein with CCT domain), floral meristem identity control protein LEAFY (LFY), and phosphatidylethanolamine-binding protein (PEBP) family protein (FT). Some AhMADS genes may be involved in flower development (such as *AG* and *AP3*), pollen development (*AGL104*), flowering time (*AGL12* and *SOC1*), root cell differentiation (*AGL12*), embryogenesis (*AGL15*), and early floral meristem (*LFY*). There were also some genes that were highly expressed in roots (*AGL21*) or acted as transcription activators that mediate floral transition in response to vernalization (*AGL24*). Detailed information on the protein annotations can be identified in **Table S9**.

### Chromosomal location and gene duplication events of peanut MADS-box TFs

**Figure 4:**
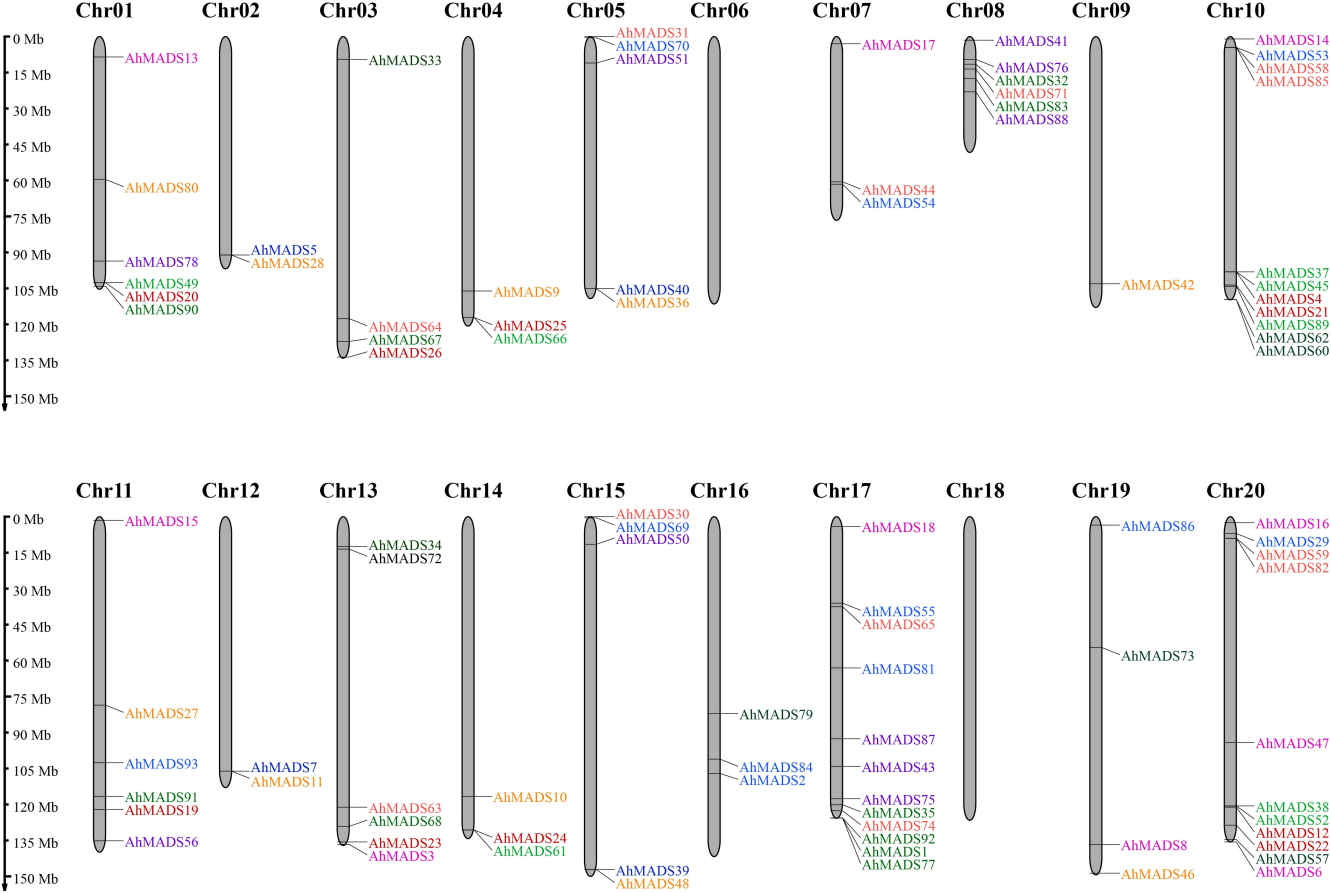
Chromosomal location of MIKC-type MADS-box proteins. In total, 93 AhMADS genes were located on 18 chromosomes. Genes from different subfamilies are shown in different colors.

The physical chromosomal location of the MADS-box genes was identified using the peanut genome database. Of the 93 genes, 58 genes were intensively distributed on 7 out of 20 chromosomes (Chr1, Chr8, Chr10, Chr11, Chr13, Chr17, and Chr20), 35 genes were randomly mapped to the 11 chromosomes, and no genes were found on chromosomes 6 and 18 (**Figure 4**). Chromosome 17 contained the maximum number of 12 genes, and chromosomes 10 and 20 had 11 genes (**Figure 4**). This observation is mainly a result of eight subfamilies (SVP, AP3, AG, AGL17, AP1, SEP, PI, and MIKC*), with most of them located on the distal telomeric parts of chromosomes 10, 17, and 20. The total number of genes in these eight subfamilies was more than 10, except for the PI subfamily (5). Furthermore, MIKC-type genes were likely to be located in the distal telomeric parts of the chromosomes (81.7% of genes). It is also noteworthy that 60% of the genes in AP3 and MIKC* subfamilies were found on Chr8 and Chr17. These results might indicate that a relatively large number of gene duplication events occur in distal telomere fragments. Similarly, the chromosomal location of MIKC-type MADS-box genes in two diploid ancestors was similar (**Figure S3**).

**Figure 5:**
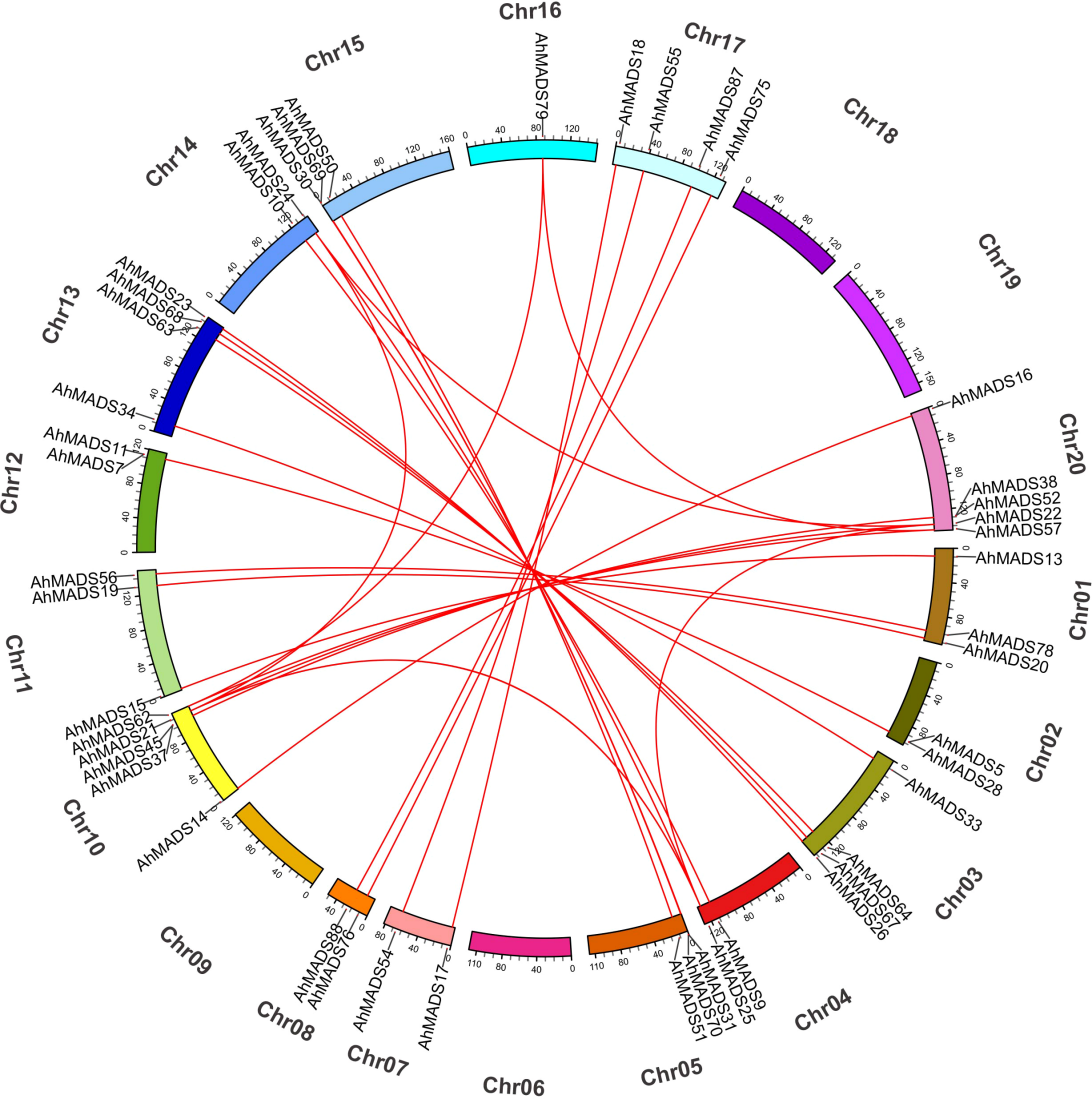
Chromosomal distributions and synteny relationships of MADS-box genes in peanut. Red lines indicate the duplicated AhMADS gene pairs in peanut.

We also analyzed gene duplication events of peanut MADS-box genes. There were three groups of two tandem duplicated genes, including AhMADS1, AhMADS92; AhMADS5, AhMADS28; AhMADS24, AhMADS61; AhMADS31, AhMADS70; AhMADS37, AhMADS45; and AhMADS38, AhMADS52. Furthermore, 29 segmental duplications were found (**Figure 5** and **Table S5**) and are visualized in **Figure 5**. In addition, five groups of tandem duplicated genes were found in two diploid ancestors (AdMADS25, AdMADS37; AdMADS34, AdMADS45; AdMADS44, AdMADS45; AiMADS14, AiMADS23; and AiMADS46, AiMADS48). Twenty-one pairs of segmental duplicated genes were identified in two diploid ancestors (**Table S5** and **Figures S4**, **5**).

### Prediction and analysis of *cis*-acting elements in the promoters of *AhMADS* TFs

**Figure 6:**
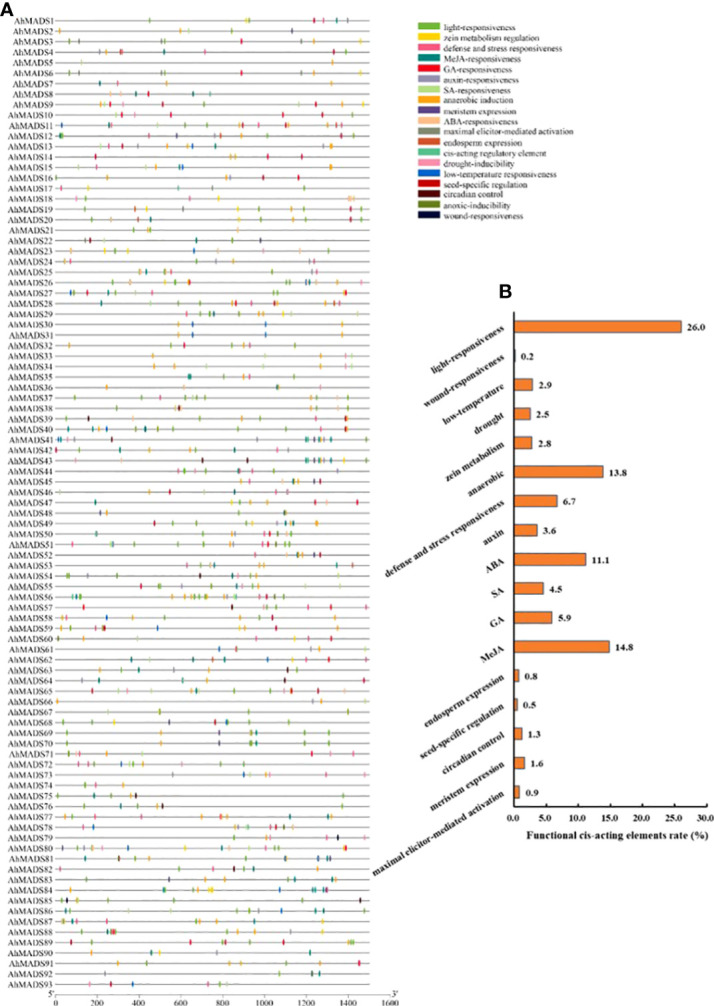
Cis-acting regulatory elements in the peanut MADS-box gene promoters. **(A)** The distribution of cis-acting elements in the promoter of each peanut MADS-box. **(B)** Various functional cis-acting elements are shown with different colors in the pie charts.

A total of 1,037 *cis*-acting elements were discovered in the 1,500-bp sequences upstream from the translational start site of each gene. Three kinds of *cis*-regulatory elements (plant growth and development, phytohormone response, and abiotic and biotic stress) were predicted in each MADS-box promoter (**Figure 6**, **Table S6**, and **Figure S6**). Nearly all the peanut MADS-box gene promoters contained *cis*-acting elements related to growth and development, plant hormones, and stress responsiveness. The number of light-responsive elements was the largest, accounting for 26% (Tifrunner) and 53% (wild type) of the total number of elements (**Figure 6**, **Figure S6**). Furthermore, a large number of MeJA-related (15%), ABA-related (11%), and anaerobic-related (14%) *cis*-acting elements were found in AhMADS promoters; a large percentage of MeJA-related (10.3%), ABA-related (7%), and anaerobic-related (14%) *cis*-acting elements were also found in AiMADS and AdMADS promoters (**Figure S6**). Some elements related to GA, SA, auxin, defense and stress response, zein metabolism, drought, and low temperature response, and a few elements related to wound response were found in promoters (**Table S6**, **Figure S6**). These findings proved that peanut MIKC-type MADS-box genes were involved in defense responses to various stresses and plant hormones.

### Expression patterns of the peanut *MADS-box genes* in different tissues

**Figure 7:**
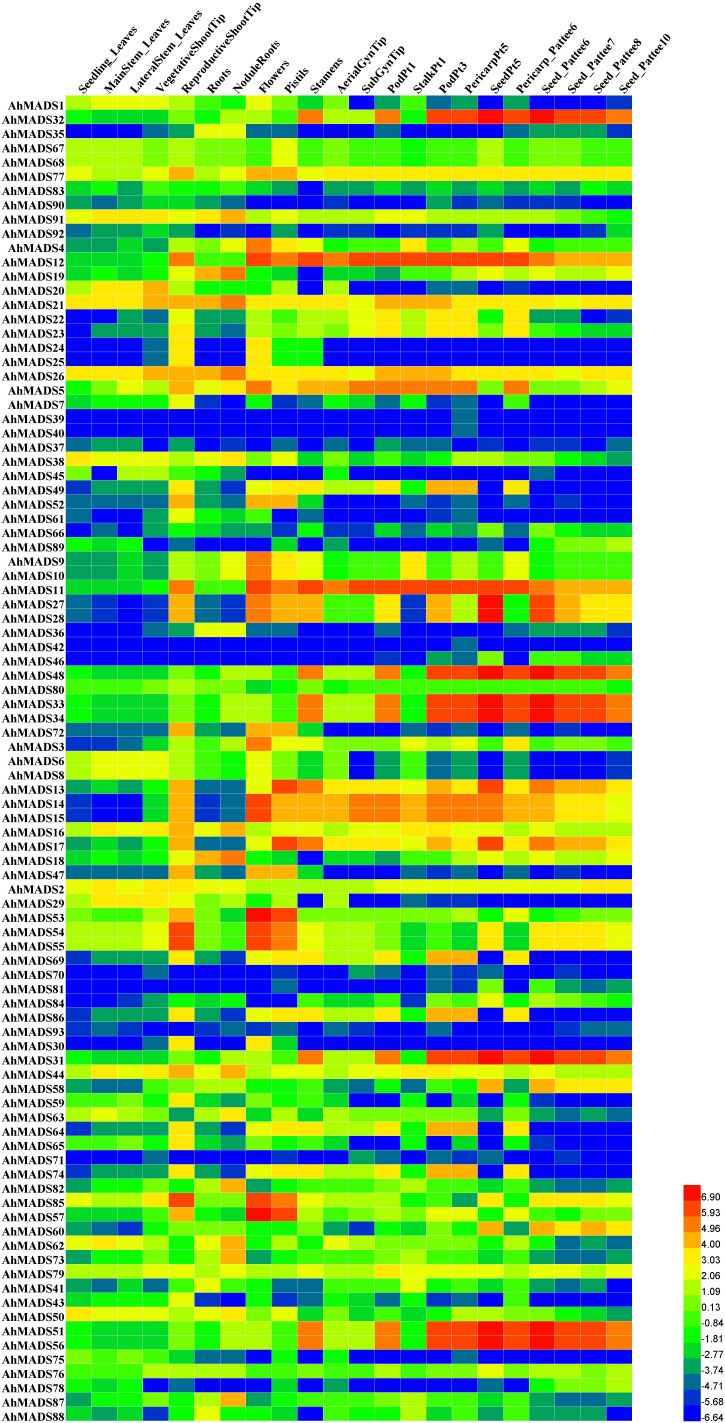
Expression profiles of MIKC-type MADS-box genes in 22 different peanut tissues. The heatmap was generated by Heml software, and the fragments per kilobase of transcript per million fragments (FPKM) values of peanut MADS-box genes were log2-transformed. The red and blue colors represent the maximum and minimum values, respectively.

*MADS-box genes* play roles in plant growth and development. To study the expression patterns of peanut MADSs, RNA-seq data of 22 tissues were analyzed for all the identified peanut MADS-box genes (**Figure 7**, **Figure S7**). Most peanut MADS-box genes showed diverse expression profiles in different tissues. We found that 38 genes were highly expressed in flower-related tissues, including 10 genes (AhMADS5, 11, 12–17, 21, and 26) in all four tissues, namely, flowers, pistils, stamens, and gynophore tips; 5 genes (AhMADS3, 4, 9, 10, and 25) were highly expressed in flowers; 7 genes (AhMADS31–34, 48, 51, and 56) in stamens; 5 genes (AhMADS49, 64, 69, 74, and 86) in pistils and stamens; 9 genes (AhMADS47, 52–55, 57, 72, 77, and 85) in flowers and pistils; and 2 genes (AhMADS27 and 28) in flowers, pistils, and stamens. A large number of genes were specifically highly expressed in floral organs, suggesting that the above genes may have an important role in flower development. In addition, the higher expression levels of some genes were identified in roots and nodule roots, implying their functions in absorbing water and inorganic salts or root development. Certain genes, AhMADS27, 28, 31–34, 48, 51, and 56, were extremely highly expressed in seeds, suggesting their seed-related functions. The expression levels of the *MADS-box genes* in different tissues are shown in **Table S6**.

### Expression patterns of AhMADSs under abiotic stresses

**Figure 8:**
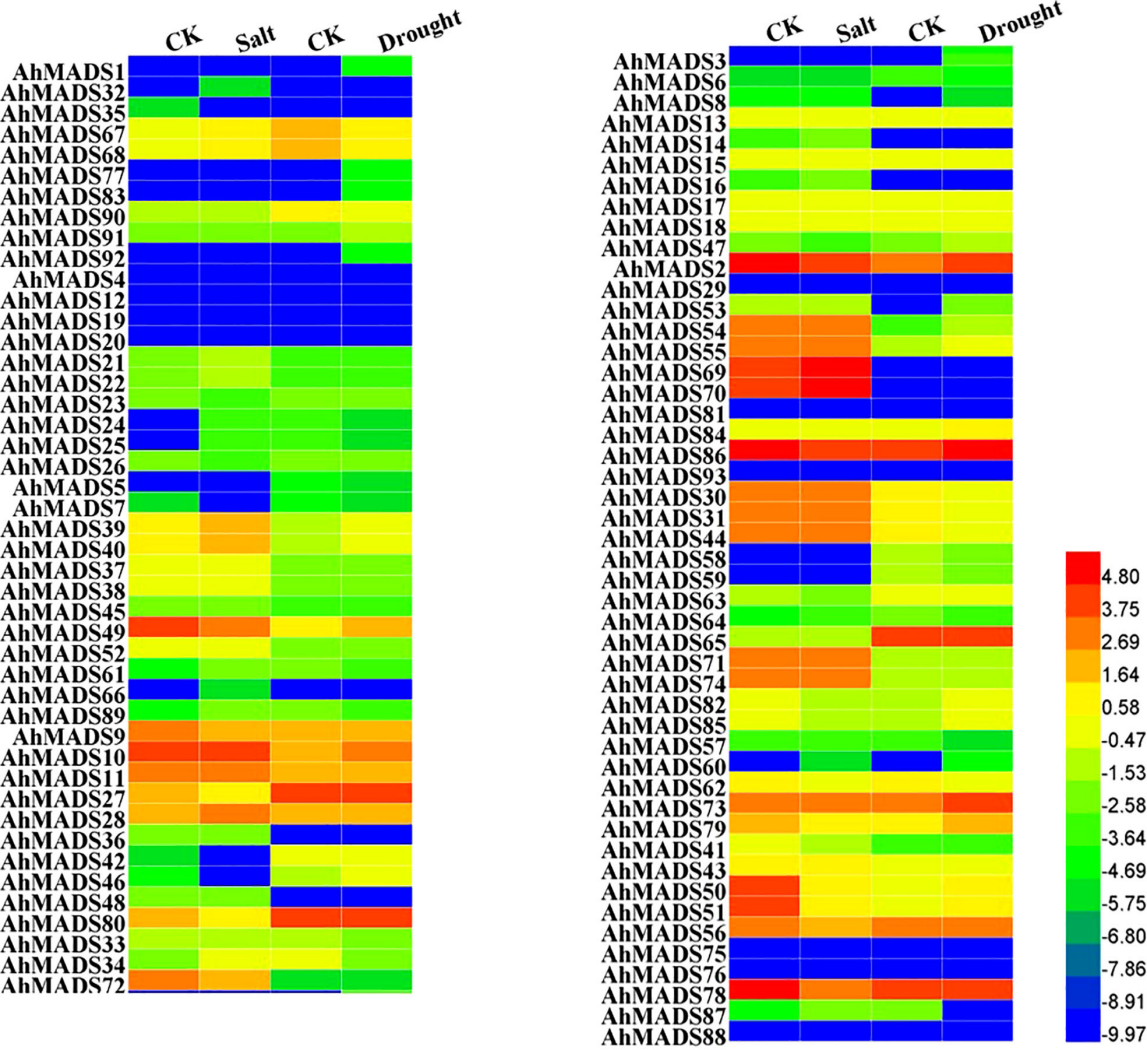
Expression levels of MADS-box genes under salt and drought stress treatments in peanut. The FPKM values of the peanut MADS-box genes were log2-transformed to create the heatmap using Heml software. The blue and red colors represent the expression levels of AhMADS genes from low to high.

To better understand the potential functions of AhMADS genes in response to abiotic stresses, we analyzed their expression levels under drought and salt treatments using RNA-seq data (**Figure 8**, **Figure S8**). As shown in **Figure 8**, peanut AhMADS genes exhibited different expression patterns under drought and salt stresses. The expression divergence was investigated by comparing the expression levels of different subfamily genes. For example, under salt treatment, the expression levels of almost all the genes from the AP3 and SVP subfamilies were downregulated, and the expression of genes from the AG, AGL17, and MIKC* subfamilies was essentially unchanged. Similarly, there was less variation in the expression of genes from the SOC1 subfamily under drought treatment. Nonetheless, multiple AhMADS genes could be induced by salt and drought stresses. Under salt treatment, the expression of most genes was downregulated, and there were also upregulated genes (**Figure 8** and **Table S8**). Consistently, similar gene expression patterns were found under drought treatment. For instance, AhMADS53 was highly induced and increased up to 175-fold, and the expression level of some genes (AhMADS6, 24, 33, and so on) was downregulated. Being different from the cultivated peanut, little AiMADS and AdMADS genes were found to be responsive to salt and drought stresses (**Figure S8**).

### Expression levels of AhMADS genes in peanut treated with abiotic stresses and hormone treatments

**Figure 9:**
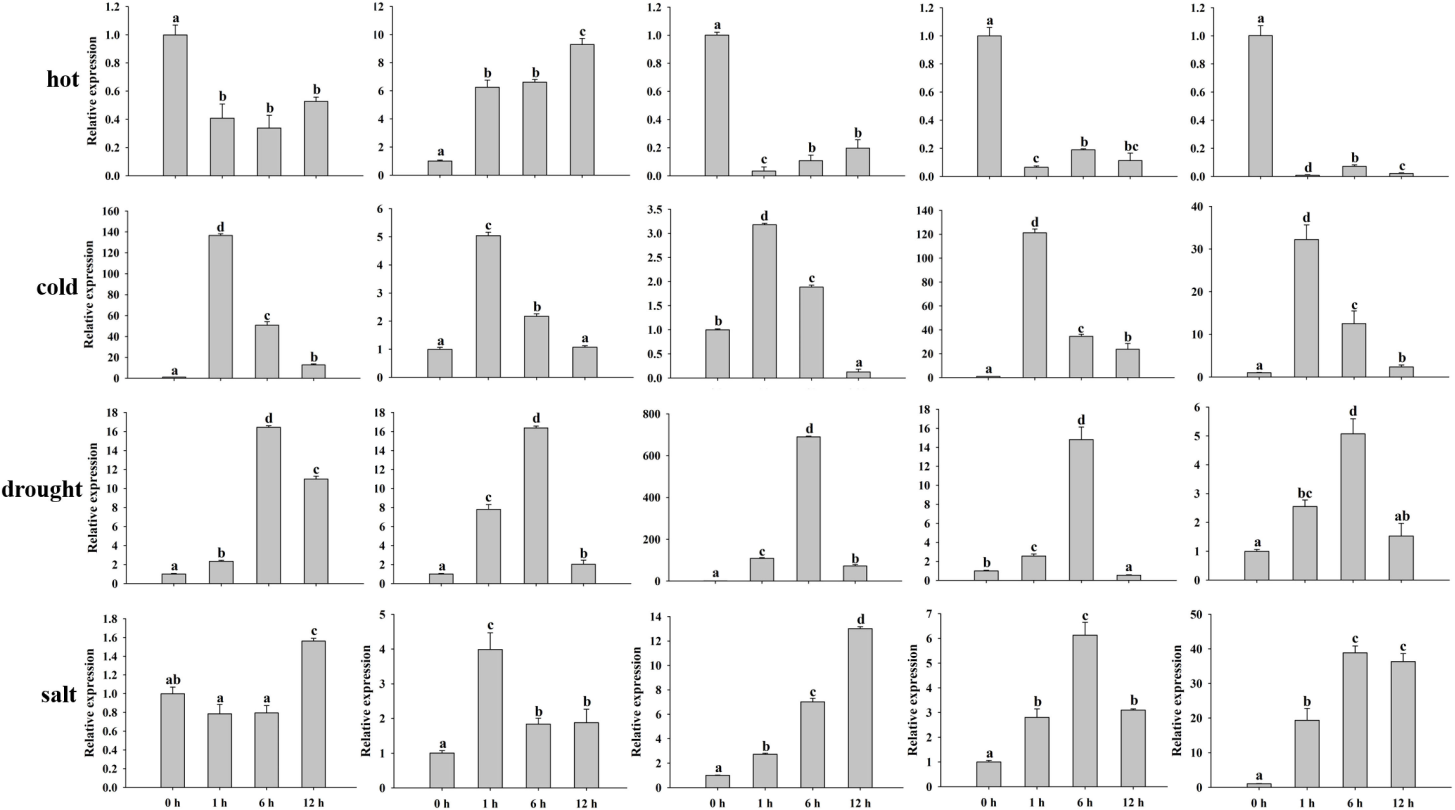
Analysis of the relative expression levels of peanut AhMADS genes by qRT-PCR. The expression profiles of five peanut AhMADS genes (AhMADS9, AhMADS21, AhMADS34, AhMADS50, and AhMADS64) under four stresses (cold, hot, drought, and salt) were validated. The data are presented as the mean ± SD (n = 3), and the values differed significantly at p < 0.05. Different letters indicate significant differences.

**Figure 10:**
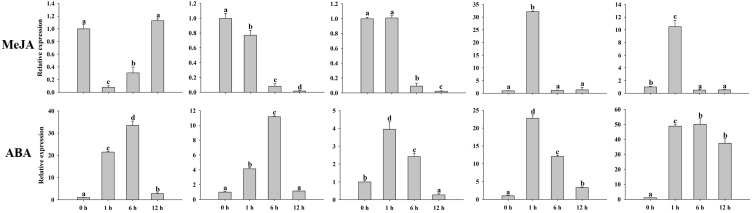
Analysis of the relative expression levels of peanut AhMADS genes by qRT-PCR. The expression profiles of five peanut AhMADS genes (AhMADS9, AhMADS21, AhMADS34, AhMADS50, and AhMADS64) under two hormone treatments (MeJA and ABA) were validated. The data are presented as the mean ± SD (n = 3), and the values differed significantly at p < 0.05. Different letters indicate significant differences.

Previous studies have proven that MADS-box genes participate in the process of plant growth and development, hormones, and various abiotic/biotic stresses. Hormone treatments (MeJA and ABA) and abiotic stresses (hot, cold, salt, and drought) were conducted to validate the peanut MADS-box relative expression levels by qRT-PCR. We investigated the relative expression of five genes (*AhMADS9*, *AhMADS21*, *AhMADS34*, *AhMADS50*, and *AhMADS64*) after 0 h, 1 h, 6 h, and 12 h of the above six treatments (**Figure 9**). The relative expression of five tested genes without *AhMADS21* was downregulated under hot treatment, and the greatest change was found for *AhMADS64*, which was decreased up to 100-fold compared with the control. Under cold, drought, salt, and ABA treatment, the relative expression of five selected genes was all upregulated, and the maximum values appeared at 1 h or 6 h. A significant increase in the relative expression of five genes after cold stress was observed; *AhMADS9* and *AhMADS50* increased above 120-fold after 1 h of treatment. Under drought treatment, the expression profiles of all detected genes were similar, and the maximum valves all appeared at 6 h. It is worth noting that the relative expression level of *AhMADS34* was increased by approximately 690-fold in response to drought stress after 6 h of treatment. After salt and ABA stress, all genes exhibited a tendency of upregulated expression, with different time points at which the maximum appeared (**Figure 10**). The five genes showed diverse expression patterns in response to MeJA treatment. Together, all the researched genes were found to respond to treatments, indicating that the members of the MADS-box gene family respond to hot, cold, salt, drought, MeJA, and ABA stress to varying degrees.

## Discussion

MADS-box TF genes play an important role in plant growth and development and environmental response. To date, the MADS-box gene family has been identified and characterized in several plants excluding peanut (Bai et al., 2019, Nardeli et al., 2018 Yi et al., 2015; Schilling et al., 2019). In this work, we identified 93 peanut MIKC-type MADS-box genes, which were divided into 12 conserved subfamilies (**Figure 1**, **Table S1**). Compared with hexaploid wheat, peanut lacks three subfamilies (FLC, *Bsister*/GGM13, and OsMADS32) (Schilling et al., 2019). Similarly, subfamilies FLC, *Bsister*/GGM13, and OsMADS32 had no MADS-box genes in autotetraploid alfalfa (Dong et al., 2021). In contrast to *Arabidopsis* and rice, peanut lost the *Bsister* subfamily, which functions in seed coat pigmentation and endothelial cell development (Nesi, 2002). *FLC*, an important repressor of floral transition, plays a key role in promoting flowering, and *flc* mutants have the phenotype of early flowering (Amasino, 1999). Its homologous genes *VRN1* and *FT*/*VRN3* in wheat and barley (Kim et al., 2009) and *Arabidopsis AGL19* (Hennig, 2008) play key roles in vernalization pathways. It is noteworthy that the flowering of peanut does not depend on vernalization or cold treatment. Approximately, 90% of the global peanuts are grown in tropical and semiarid tropical regions, and they are sensitive to temperature (Rasad et al., 1999; Hamidou et al., 2013). Thus, the loss of *FLC* genes may similarly be explained by adaptation and/or acclimatization.

The exon−intron gene structure is relatively conserved throughout evolution (Rogozin et al., 2003). Phylogenetic and gene structure analyses showed that most of the MIKC-type MADS-box genes in the same subfamily have a similar exon−intron structure (**Figure 2**). This indicated that the gene structure and functions of AhMADSs might be conserved during evolution. Different gene structures (gain or loss introns) were also identified in AhMADS genes, suggesting that evolution might affect not only gene function but also gene structure (Babenko et al., 2004; Penny, 2007). The diversiform gene structure is conducive to their various functions (Mattick and John, 1994). In peanut, motif 1 and motif 2 encode the typical MADS-box TF (SRF), which is extremely conserved among AhMADSs (**Figure 2**). The K-domain, the other conserved domain in the MADS-box gene family, consists of motif 3. Generally, the K domain only exists in the MIKC^C^ subfamily (Parenicová et al., 2003). Currently, *AhMADS32* and *AhMADS35* of peanut have been found in MIKC^*^ families that contain K motifs. One gene, *ZjMADS51*, was also found to contain a K motif in Chinese jujube (Zhang et al., 2017). MIKC-type MADS-box proteins without K domains are still able to bind DNA and function as full domain proteins (Kaufmann et al., 2005). However, the absence of the K domain results in functional impairment of the TaSEP1-A2 protein without protein−protein interactions (Shitsukawa et al., 2007). Whether the diversity of the MADS-box domain influences the function of MADS-box genes requires further study.

We found that 81.7% of peanut MIKC-type genes tended to be located in distal telomeric segments (**Figure 3**). The distal regions of chromosomes have been considered targets of recombination, and duplicated genes were found more frequently in this region (Salse et al., 2008; Choulet et al., 2014). The rapid evolution of genes lies within the higher recombination at the distal regions (Chen et al., 2018). In plants, genes related to stress response and environmental stimulation have been found to be generally located in distal regions of chromosomes (Ramirez-Gonzalez et al., 2018; Wang, 2020). Remarkably, all the tandem duplications (15 pairs) and most of the segmental duplications (25 out of 29 pairs) were found to be located in distal telomeric segments of the chromosome (**Figure 3** and **Table S5**). These duplicated genes were distributed in almost all subfamilies except AGL12 (only one gene), with more tandem duplicated genes in subfamilies SEP, AP1, and SVP as well as segmental duplicated genes in subfamilies AG, AP3, and SEP. It is noteworthy that these duplicated genes might be beneficial to generate new functions and contribute to adaptation to the complex environment (2018). In addition, we found that the number of peanut MIKC-type MADS-box genes (93) was greater than that in *Arabidopsis* (44) (Parenicová et al., 2003), *Medicago sativa* L. (45) (Dong et al., 2021), rice (34) (Arora et al., 2007), and soybean (72) (Shu et al., 2013), but less than tobacco (94) (Bai et al., 2019) and wheat (201) (Schilling et al., 2019). This might be explained by its allotetraploid genome, with genomes as large as 2.8 Gb, which experienced hybridization between *Arachis duranensis* and *Arachis ipaensis* during evolution. Gene duplication is the main driving force in expanding gene families in plants, and new genes and new functions were produced during plant evolution (Bowers et al., 2003; Cannon et al., 2004).

A majority of the MADS-box genes have been shown to respond to stress and phytohormone treatments. Consistent with previous results, we found that abundant *cis*-acting regulatory elements in gene promoters were related to abiotic/biotic (anoxic conditions, cold, drought, wounding, zein metabolism, etc.) and hormones (MeJA, ABA, GA, SA, auxin, etc.). It is estimated that ABREs were the most abundant element, followed by GT1-motif elements. This result was not completely similar to the experimental discovery of MADS-box genes in Eudicots and the gene family analysis in alfalfa (Liu et al., 2018; Dong et al., 2021). In fact, the only difference is that the number of GT1-motif elements is larger than that of ABREs. It has been reported that ABREs are *cis*-acting elements involved in ABA responsiveness, regulating the ABA-related gene expressions in plants (Yamaguchi-Shinozaki and Shinozaki, 2006). Our finding was consistent with previous studies that show that MADS-box genes were important and necessary for the responses to various stresses (Duan et al., 2015; Jian et al., 2017; Dong et al., 2021), and further exploration of the potential biological functions of MADS-box genes is needed.

Functional predictions of the MADS-box genes can be conducted by investigating their gene expression patterns; hence, we analyzed the RNA-seq data for 22 peanut tissues. Data showed that the expression profiles of MADS-box genes were tissue-specific (**Figure 6**), which were closely related to gene function. For instance, *Arabidopsis AGL12* plays a potential role in flowering transition, and its mutants *xal1*(*agl12*) are able to grow under long days with late-flowering phenotypes (Tapia-Lopez et al., 2008). FLC acts as a repressor in *Arabidopsis* flowering time, while the FLC subfamily genes were absent in peanut. We have discussed probable causes of missing data, and more analyses are needed to explore the evolutionary importance of these genes. The *STK* gene, a member of the AG subfamily in *Arabidopsis*, is essential for ovule and seed development, and its homologous gene *SHELL* in oil palm regulates oil yield (Rajinder Singh et al., 2013). The homologous genes of AG subfamily members (*AhMADS3* and *AhMADS13*–*18*) in peanut were highly expressed in seeds, indicating that they may participate in the regulation of the development and oil production of seeds. Thereby, it can be inferred from the fact that homologous genes have similar functions. SEP1/2/3 genes play key roles in *Arabidopsis* floral organ (petals, stamens, and carpels) development (Pelaz et al., 2000), and triple mutant *Arabidopsis* plants produce flowers without sepals. The tomato AGAMOUS-LIKE1 (TAGL1) gene, which belongs to the SEP subfamily, is necessary for fruit development (Vrebalov et al., 2009). In rice, the SEP homologues *OsMADS5* and *OsMADS34* function in spikelet and flower development (Zhu et al., 2021). To sum up, the tissue-specific analysis provides new and deep insights into the functional characterization of AhMADSs.

The MADS-box is a multifunctional gene family that has been proven in several plants. The analysis of *cis*-acting elements, RNA-seq, and qRT−PCR data in our study further confirmed this point. We found that AhMADSs not only participated in plant growth and development (**Figures 6**, **7**) but were also involved in the response to biotic and abiotic stresses (**Figures 6**, **8**, **9**). For maize, the expression of *ZZM7-L* was upregulated under cold, NaCl, and drought treatments and downregulated under ABA treatment (Zhang et al., 2012). In addition, its overexpressed *Arabidopsis* plants had a lower germination rate than wild type with NaCl and mannitol stresses, demonstrating that *ZZM7-L* might be a negative gene in response to abiotic stresses (Zhang et al., 2012). It was also corroborated that *CaMADS* could function in response to salt, cold, and osmotic stress, and overexpressed *CaMADS* led to enhanced resistance to these stresses in *Arabidopsis* (Chen et al., 2019). Furthermore, rice *OsMADS26* negatively regulates resistance to drought tolerance (Khong et al., 2015). *Arabidopsis* AGAMOUS-LIKE22 (AGL22) is involved in the transition from vegetative state to flowering, and affects photosynthetic rates and water use (Bechtold et al., 2016). More importantly, AGL22 plays a vital role in linking changes in primary metabolism and the initiation of drought stress responses (Bechtold et al., 2016). Although previous studies have paid attention to the abiotic functions of some MADS-box genes, only very few studies about the function of MADS-box genes could be found in peanut. In this study, the results of abiotic stress treatments were consistent with the above cited trials. The expressions of five peanut genes (AhMADS9, 21, 34, 50, and 64) increased in response to three abiotic treatments (cold, drought, and salt) (**Figure 9**). Collectively, this analysis further proved that MADS-box genes are a multifunctional gene family involved in the regulation of abiotic stress in peanut, and the function network requires further investigation.

## Conclusion

In this study, 186 MIKC-type MADS-box genes in the peanut genome were identified and divided into 12 subfamilies. The analysis of phylogenetic relationships, gene structures, and protein motifs revealed that most peanut MADSs were relatively conserved. A large number of AhMADS genes were located on 7 out of 20 peanut chromosomes. Gene duplication played important roles in the expansion of the MADS-box gene family. The results of *cis*-acting elements and the expression patterns of tissues and different stresses (RNA-seq and qRT−PCR data) revealed that MIKC-type MADS-box genes in peanut were involved in development, hormones (MeJA, ABA, etc.), and abiotic stresses (cold, heat, salt, drought, etc.). The prediction of the interaction network provided clues for finding the related mechanisms of genes. Our study will be helpful for further functional research and exploration of the mechanism of action.

## Data availability statement

The original contributions presented in the study are included in the article/**Supplementary Material**. Further inquiries can be directed to the corresponding author.

## Author contributions

YM, CL and SS conceived and designed the study. YM performed the experiments and wrote the manuscript. QS helped the bioinformatics analysis. CuY provided the plant materials. JW, XZ, CaY and QW revised the paper. All authors contributed to the article and approved the submitted version.

## Funding

This work was supported by the Innovation Project of SAAS (CXGC2021B33, CXGC2022A03, CXGC2022A21), National Natural Science Foundation of China (31901506, 32072328), the Taishan Scholar Project of Shandong Province (ts201712080), General project of Natural Science Foundation of Shandong Province (ZR2021MC124), the Agro-industry Technology Research System of Shandong Province (SDAIT-04-02), and the Shandong Elite Variety Project (2020LZGC001).

## Acknowledgments

We are grateful to all the colleagues who provided helpful databases and useful assistance in our laboratory.

## Conflict of interest

The authors declare that the research was conducted in the absence of any commercial or financial relationships that could be construed as a potential conflict of interest.

## Publisher’s note

All claims expressed in this article are solely those of the authors and do not necessarily represent those of their affiliated organizations, or those of the publisher, the editors and the reviewers. Any product that may be evaluated in this article, or claim that may be made by its manufacturer, is not guaranteed or endorsed by the publisher.
