# Supplementary figures and images for: MIKC-type MADS-box transcription factor gene family in peanut: Genome-wide characterization and expression analysis under abiotic stress
Source: Front Plant Sci. 2022 Oct 20;13:980933. doi: 10.3389/fpls.2022.980933 (PMC9631947; doi:10.3389/fpls.2022.980933)

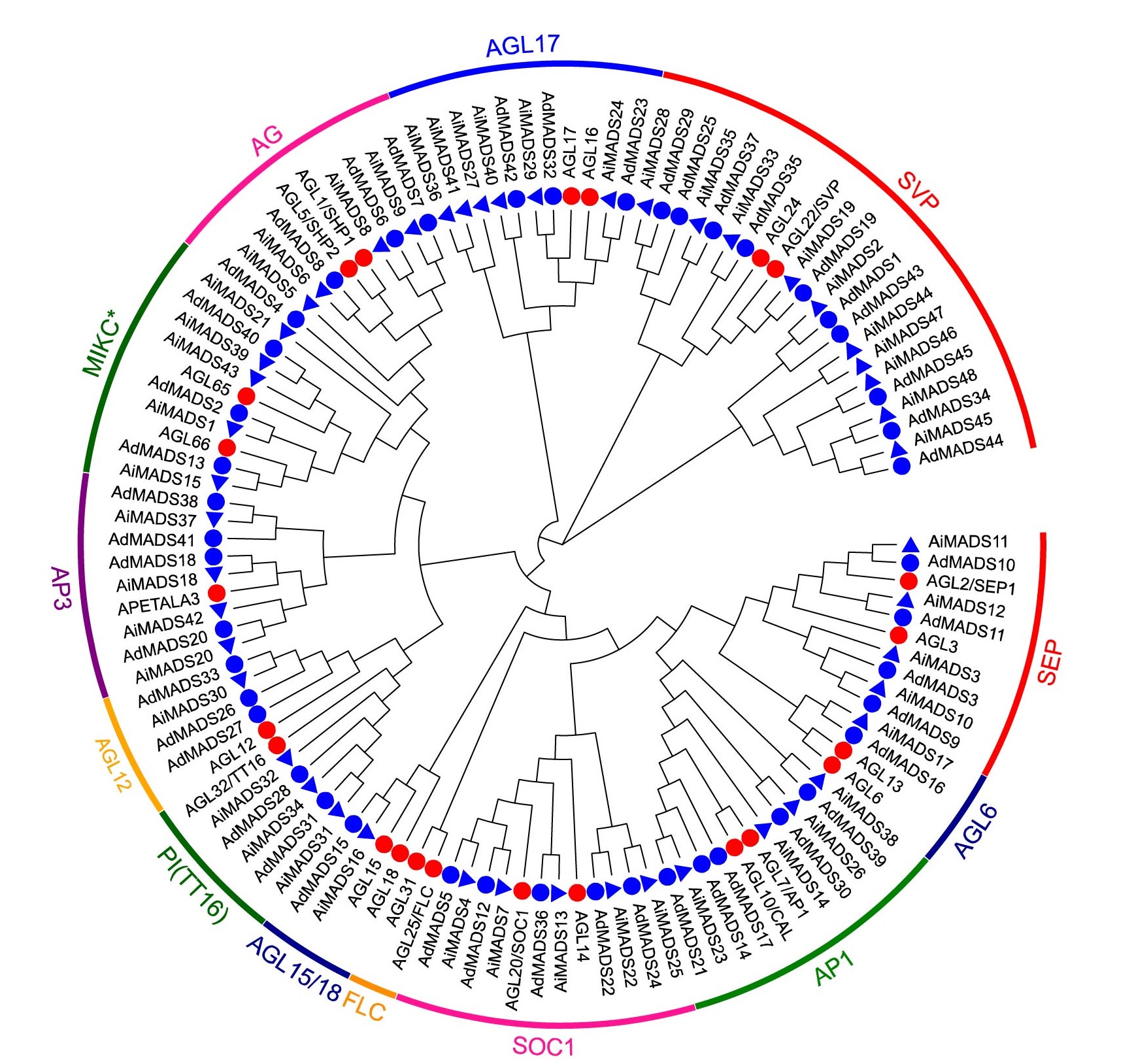

Supplement: Supplementary file 1 [file Image_1.jpeg]

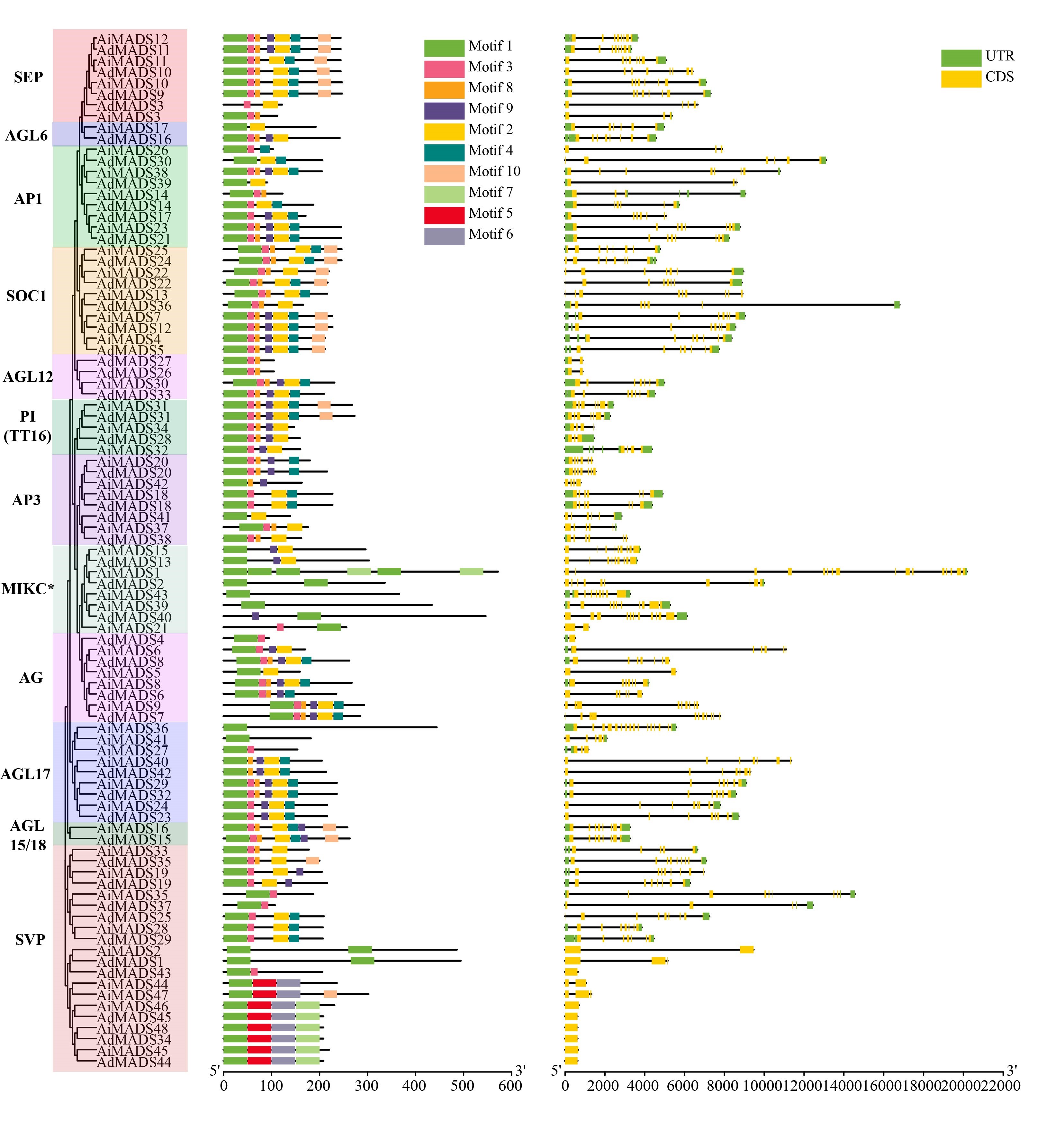

Supplement: Supplementary file 2 [file Image_2.jpeg]

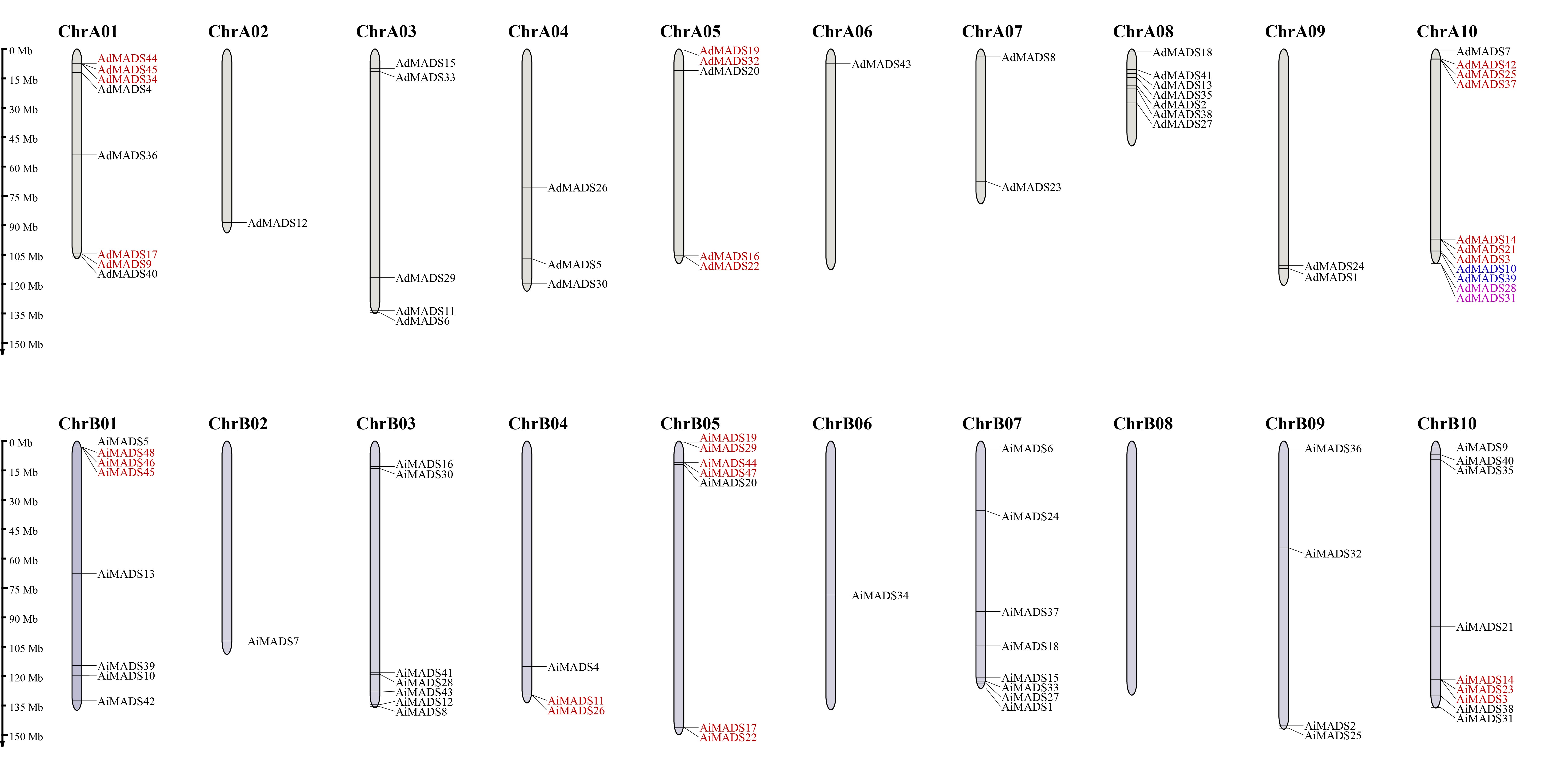

Supplement: Supplementary file 3 [file Image_3.jpeg]

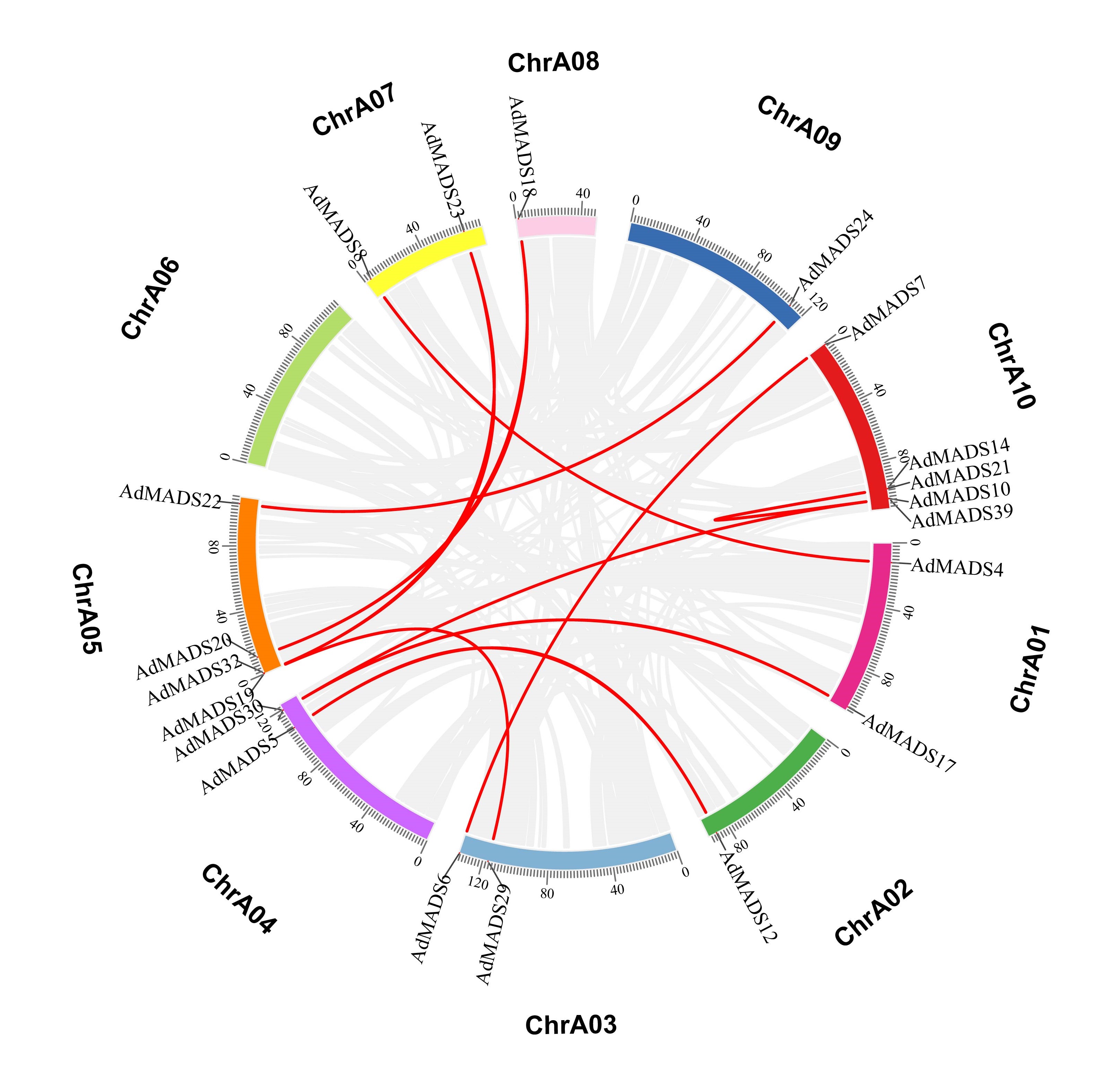

Supplement: Supplementary file 4 [file Image_4.jpeg]

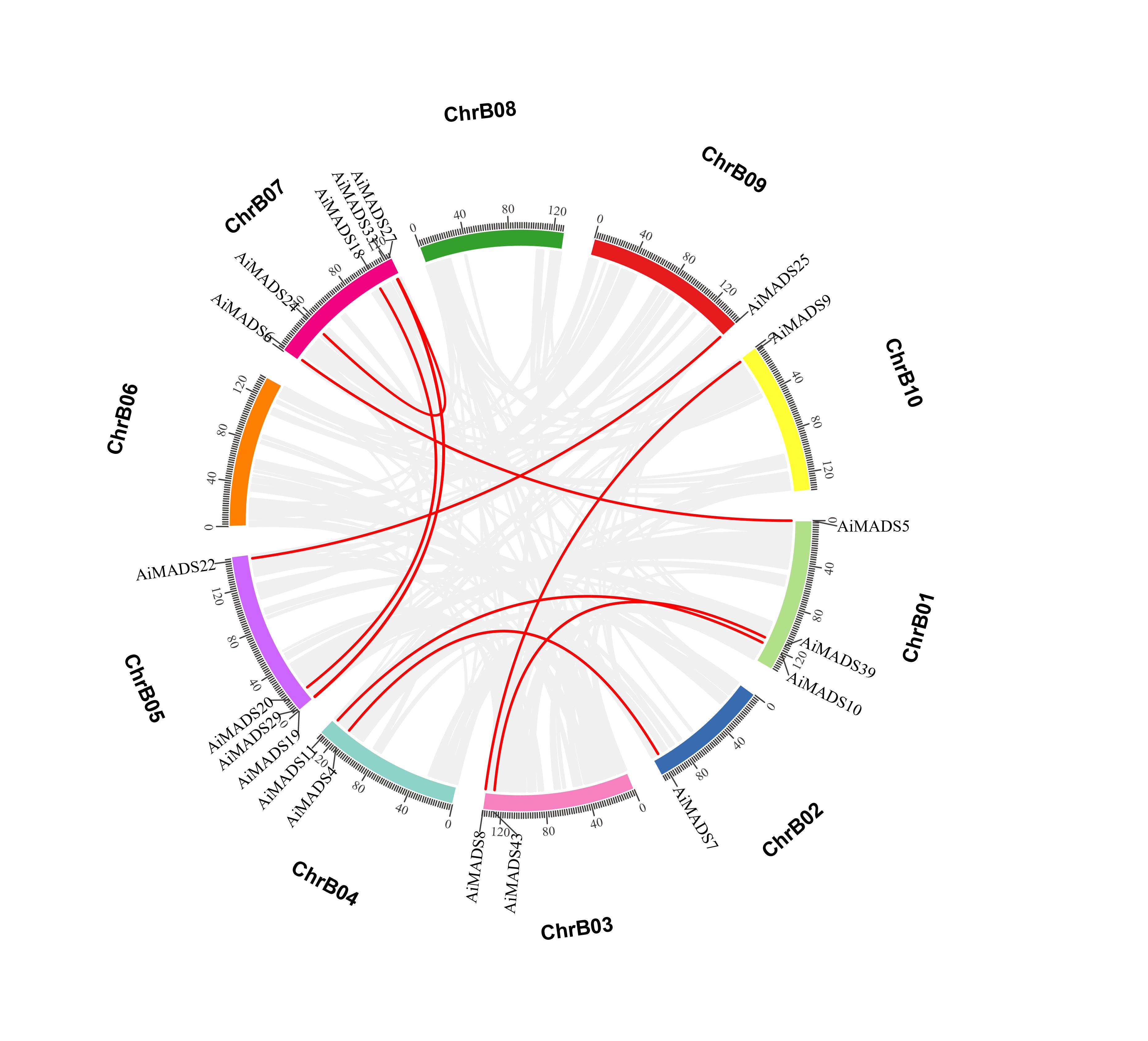

Supplement: Supplementary file 5 [file Image_5.jpeg]

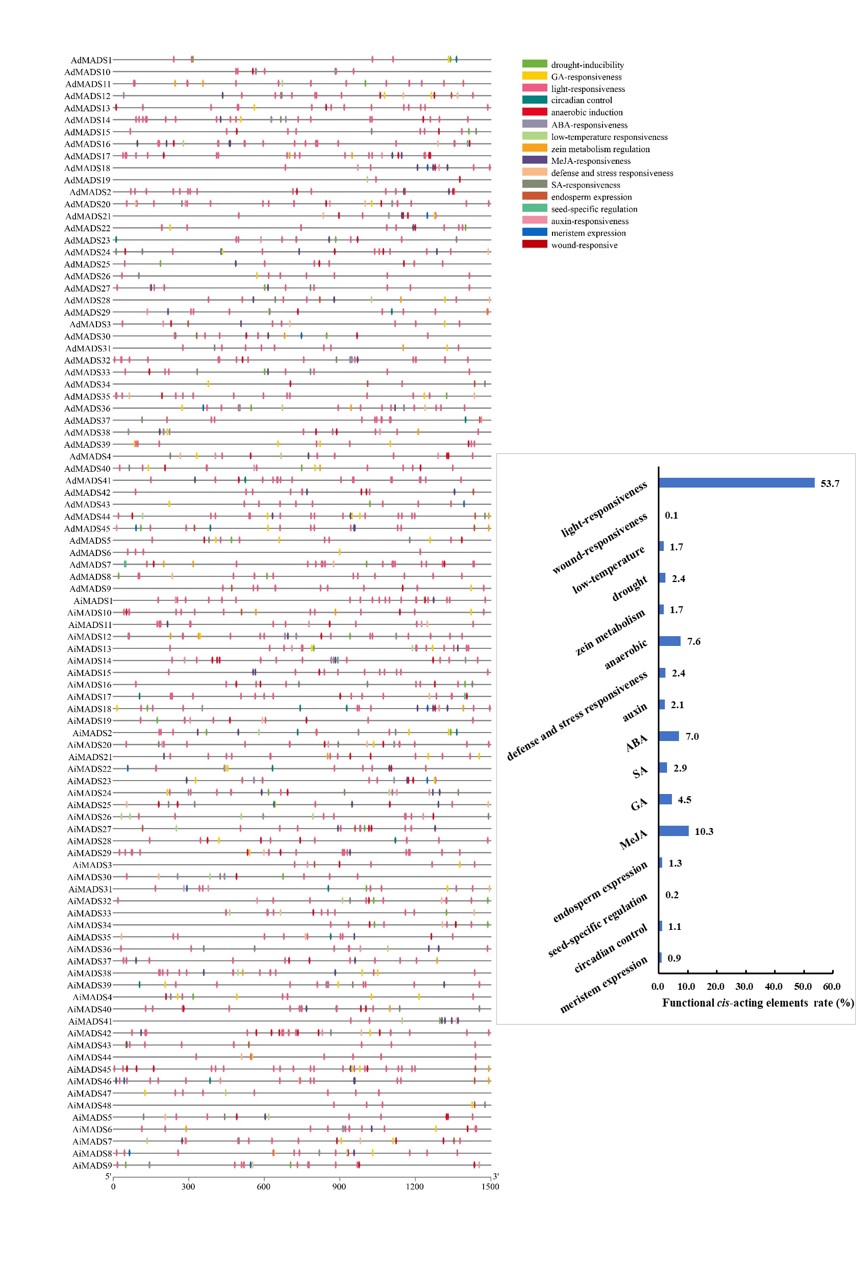

Supplement: Supplementary file 6 [file Image_6.jpeg]

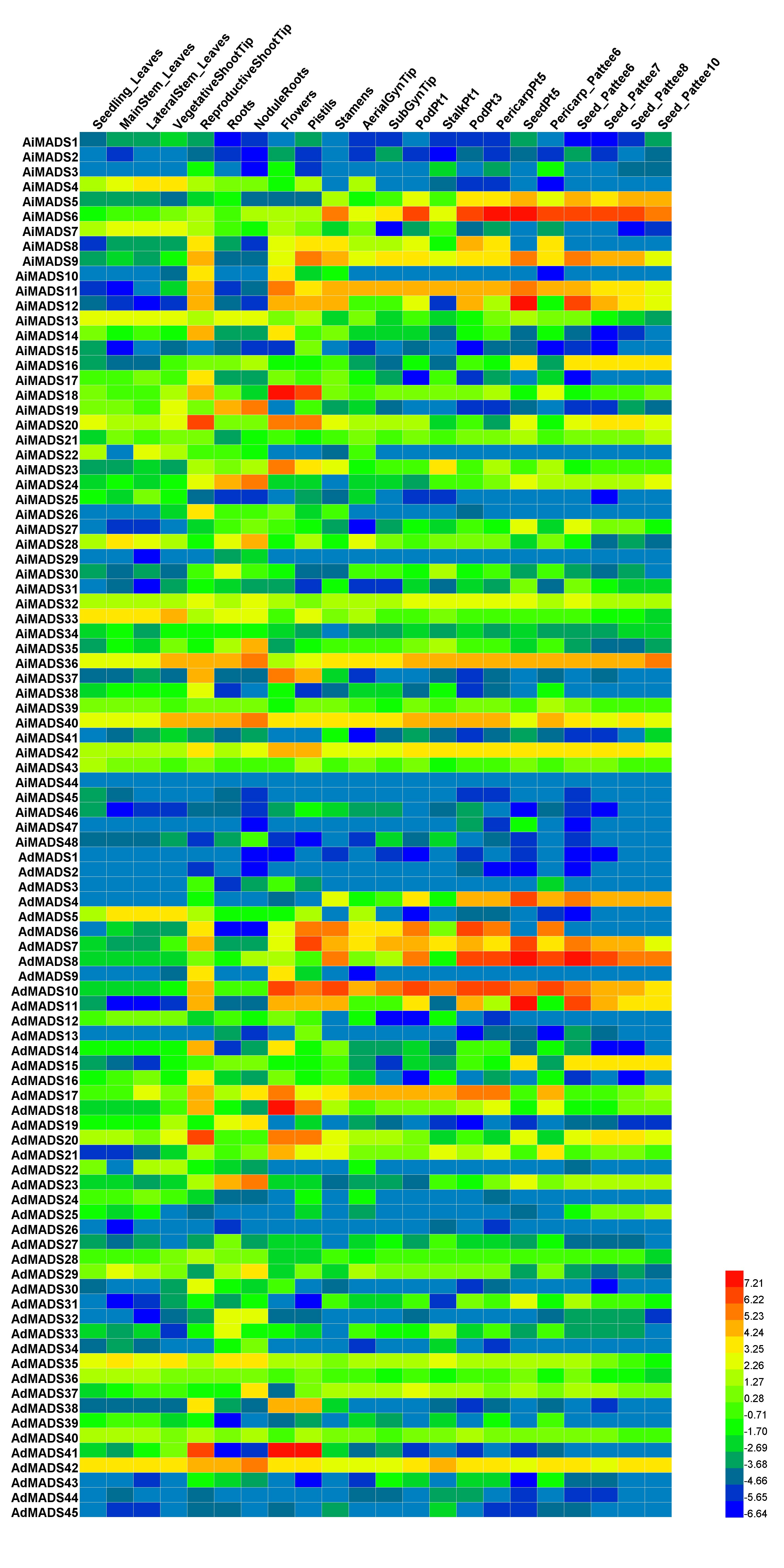

Supplement: Supplementary file 7 [file Image_7.jpeg]

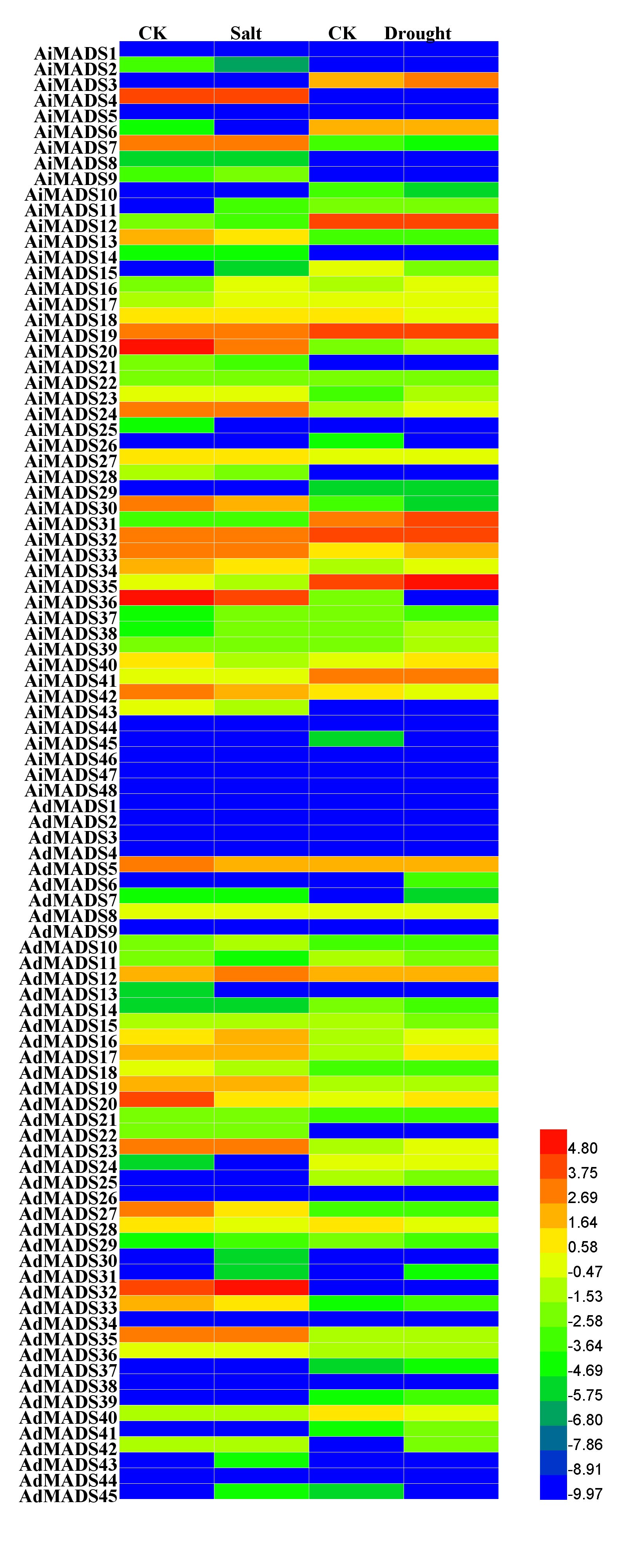

Supplement: Supplementary file 8 [file Image_8.jpeg]
